# Interfacial electronic coupling in MX–MX (M = Cu, Ag, and Au; X = Cl, Br, and I) van der Waals heterostructures for photocatalytic water splitting and broadband optoelectronic applications

**DOI:** 10.1039/d6ra05749g

**Published:** 2026-08-28

**Authors:** Ayesha Zahid, M. Idrees, Zijing Lin, B. Amin

**Affiliations:** a Department of Physics, Abbottabad University of Science & Technology Abbottabad 22010 Pakistan binukhn@gmail.com zjlin@ustc.edu.cn; b School of Chemistry and Chemical Engineering, Shandong University Jinan 250100 China; c Department of Physics, University of Science and Technology of China Hefei 230026 China

## Abstract

The structural, electronic and optoelectronic properties of two-dimensional (2D) van der Waals heterostructures (vdWHs) can be effectively modulated for high-performance energy applications. Here, we use first-principles density functional theory to systematically investigate the structural stability, electronic properties, interfacial charge coupling, and optical and photocatalytic performance of MX–MX (M = Cu, Ag, and Au; X = Cl, Br, and I) vdWHs. The optimized vdWHs exhibit favourable energetic stability, with type-I and type-II semiconducting nature and pronounced interfacial electronic coupling, promoting the efficient separation of photogenerated charge carriers. Charge redistribution analysis further confirms intrinsic interlayer charge transfer and the formation of built-in electric fields across the interface of MX–MX vdWHs. The calculated optical spectra show a strong broadband optical absorption extending from the near-infrared to ultraviolet regions, accompanied by an enhanced dielectric response and high absorption coefficients, indicating the excellent light-harvesting capability of MX–MX vdWHs. Band edge alignment in comparison to water redox potentials demonstrates that AgBr-CuI vdWHs possess a suitable energetic alignment for overall photocatalytic water splitting at pH = 0, while the other vdWHs are mainly favourable for photocatalytic oxidation reactions. Our findings highlight MX–MX vdWHs as a promising candidate for solar-driven hydrogen production, photocatalysis, and next-generation broadband optoelectronic devices.

## Abstract 2

Photocatalytic response of AgBr-CuI vdWHs, with the black and red lines showing the redox potential for full water splitting.

## Graphical abstract

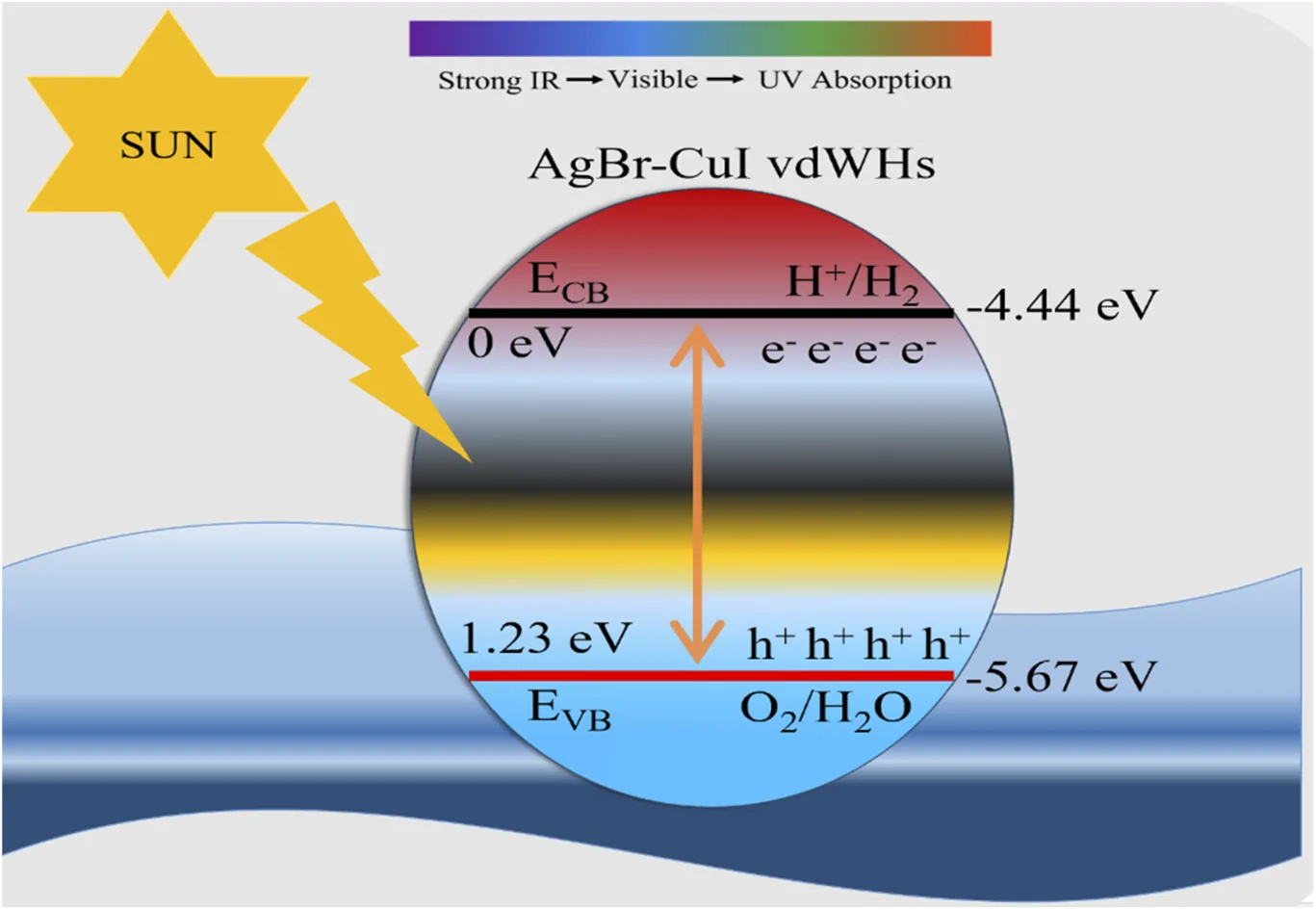

## Introduction

Recently, the growing global energy crisis and environmental degradation caused by fossil fuel consumption have accelerated search for advanced materials among researchers working on sustainable energy technologies. Among various renewable energy sources, semiconductor-based photocatalytic water splitting has emerged as a promising strategy for clean hydrogen (H_2_) production through solar-driven redox reactions.^1–3^ In this method, upon light irradiation, electrons are excited from the valence band maximum (VBM) to the conduction band minimum (CBM), generating electron–hole pairs that participate in H_2_ and oxygen (O_2_) evolution reactions. Therefore, the optical properties of two dimensional materials have attracted immense attention due to their significant role in light–matter interactions and optoelectronic performance.^4,5^ Optical parameters such as absorption coefficient, dielectric function, refractive index, reflectivity, and optical conductivity provide crucial insights into the electronic excitation and photon absorption behaviour of materials, which helps identify materials that are highly promising for next-generation optoelectronics and energy-related applications.^6–8^

Since the isolation of graphene,^9^ two-dimensional (2D) materials have revolutionized nanomaterials research due to their extraordinary physicochemical properties and reduced dimensional quantum confinement effects.^10,11^ In contrast to their bulk counterparts, 2D materials exhibit highly tuneable electronic structures, superior surface-to-volume ratios, enhanced carrier transport properties, and remarkable light–matter interactions, enabling their extensive applications in spintronics, optoelectronics, nanoelectronics, photocatalysis, sensing technologies, and energy storage devices.^10,12–15^ The zero-band gap nature of graphene significantly restricts its applicability in semiconductor-based optoelectronic and photocatalytic systems, which require efficient charge separation and finite band-gap modulation.^16,17^ Consequently, substantial research efforts have been directed toward the exploration of many alternative 2D semiconductor materials possessing intrinsic band gaps and favourable optoelectronic characteristics.^18–20^ In addition to these 2D monolayers, metal halides have attracted enormous scientific attention due to their highly tuneable mechanical, electronic, magnetic as well as optical properties, and they have substantially broadened the family of emerging 2D materials. Particularly, group-11 transition-metal halide monolayers, MX (M = Cu, Ag, and Au; X = Cl, Br, and I), exhibit remarkable thermodynamic and dynamical stability with a direct wide band gap nature and exceptionally high carrier mobility, which makes them attractive for ultraviolet optoelectronic devices, photocatalytic water-splitting systems and solar energy applications.^21–23^ Moreover, their strong optical absorption response and favourable charge transport nature highlight the technological potential of these monolayers for advanced optoelectronic and energy conversion applications.^21,23^

To modulate and enhance the intrinsic properties of 2D materials, various engineering strategies have been extensively explored.^24–27^ Among the different strategies, van der Waals heterostructure (vdWH) formation has emerged as a highly versatile platform, where the atomically thin layers are vertically stacked through weak interlayer vdW interactions, enabling novel interfacial electronic and optoelectronic phenomena.^28–31^ Particularly, the type-II band alignment in these vdWHs promotes the efficient spatial separation of photogenerated charge carriers and suppresses electron–hole recombination, making them highly promising for optoelectronic, nanoelectronic, photovoltaic, photocatalytic and solar-energy-conversion applications.^19,32,33^

Motivated by the remarkable physicochemical properties of MX (M = Cu, Ag, and Au; X = Cl, Br, and I) monolayers, we systematically designed and investigated MX–MX vdWHs, such as AgBr-AuBr, AgBr-AuI, AgBr-CuI, AgCl-AuBr, AgCl-AuI and AuBr-CuI vdWHs, using density functional theory calculations. Detailed structural analysis confirmed that the most energetically favourable stacking configurations possessed excellent thermodynamic and thermal stability. Furthermore, the electronic band structures, interfacial charge redistributions, charge density differences and work functions of MX–MX vdWHs were comprehensively examined to elucidate their interlayer coupling and charge transfer behaviour. Finally, the optoelectronic and photocatalytic response of these systems was systematically explored, which revealed their strong potential for advanced nanoelectronics and next-generation optoelectronic and photocatalytic devices.

## Computational details

First-principles calculations were used in the framework of density functional theory (DFT), implemented in the Vienna *ab initio* simulation package (VASP).^34,35^ The exchange-correlation energy and electron–ion interactions were investigated using the generalized gradient approximation (GGA) combined with the projector-augmented wave (PAW) method, carried out within the Perdew–Burke–Ernzerhof (PBE) functional.^36,37^ A kinetic energy cutoff of 500 eV was applied to the plane-wave basis set. All vdWHs were fully optimized until the forces on each atom were below 0.001 eV Å^−1^ and the total energy converged to 10^−6^. The first Brillouin zone (BZ) was applied using a *Γ*-centered 12 × 12 × 1 *k*-point mesh, and a vacuum layer of about 30 Å along the *z*-direction was added to eliminate artificial interactions between periodic layers on these systems. Long-range dispersion forces were incorporated using the Grimme DFT-D3 correction scheme to ensure an accurate representation of interlayer vdW interactions.^38^ The Heyd–Scuseria–Ernzerhof hybrid functional (HSE06) was used to obtain more reliable band gaps and electronic band structures for MX–MX vdWHs.^39^ Thermal stability was investigated using *ab initio* molecular dynamics (AIMD) simulations in the canonical ensemble using a Nosé thermostat at room temperature, and the binding energy was calculated using *E*_b_ = *E*_MX−MX_ − *E*_MX_ − *E*_MX_.^40^ The work function (*∅*) and charge density difference were determined using^43^*∅* = *E*_vac_ − *E*_F_ and *ρ* = *ρ*_MX−MX_ − *ρ*_MX_ − *ρ*_MX_^44^ for charge transfer, respectively. Further, optical properties were calculated from the frequency-dependent complex dielectric response using the Bethe–Salpeter equation (BSE) approach.^41^ Finally, we examined the photocatalytic properties of MX–MX vdWHs for solar-driven hydrogen evolution using Mulliken electronegativity (*χ*) analysis for different pH values.^42^

## Results and discussion

The structural parameters obtained for the fully relaxed isolated AgBr, AuBr, AgCl, AuI and CuI monolayers are 4.35 Å, 4.268 Å, 4.40 Å, 4.48 Å, and 4.21 Å, respectively, in excellent agreement with previously reported theoretical and experimental data, validating the accuracy and reliability of our employed first-principles computational methodology.^21–23,43^ Furthermore, these MX monolayers possess a closely matched hexagonal symmetry with minimal lattice mismatch, about 4%, which is a fundamentally important criterion for the fabrication of high-quality vdWHs. This negligible lattice mismatch also indicates the potential feasibility of experimental realization through advanced epitaxial growth and layer-by-layer deposition techniques.^44,45^

**Fig. 1:**
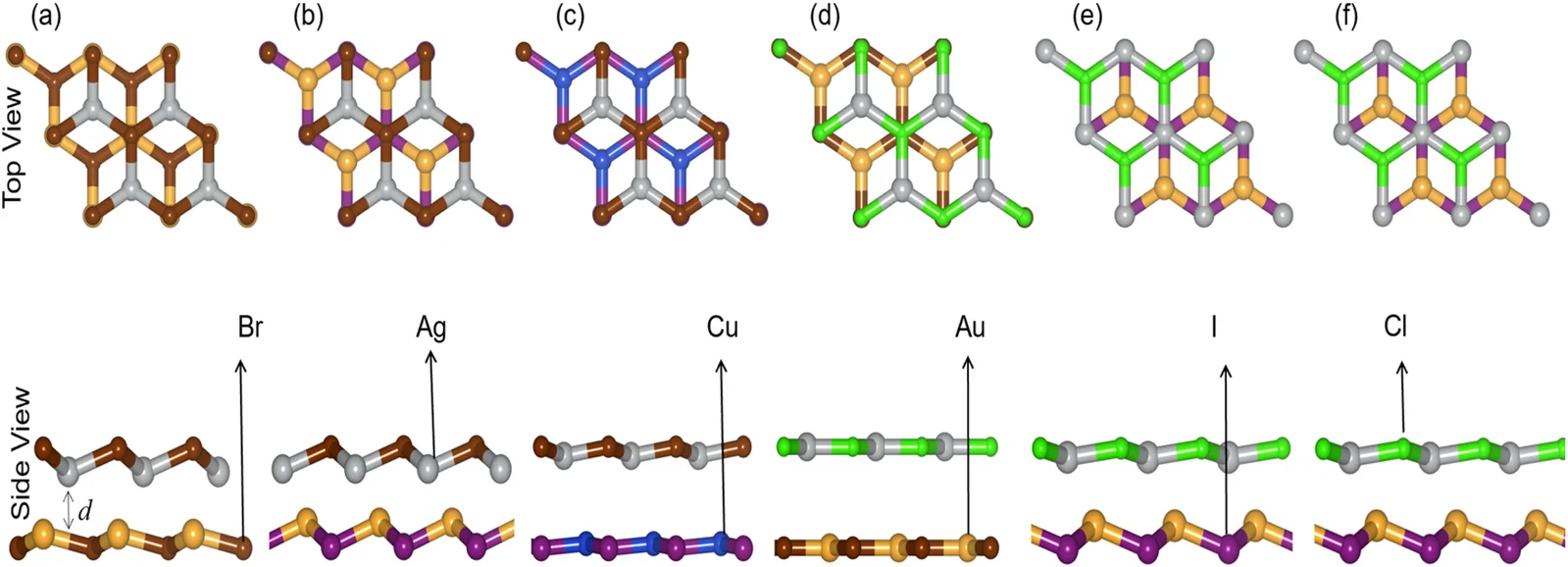
Top and side views of the most favourable stacking configurations of the (a) AgBr-AuBr, (b) AgBr-AuI, (c) AgBr-CuI, (d) AgCl-AuBr, (e) AgCl-AuI and (f) AuBr-CuI vdWHs, with the legends of the atoms mentioned in the figure.

To construct the MX–MX vdWHs, fully optimized geometries of the parent monolayers were vertically stacked using primitive 1 × 1 × 1 unit cells owing to their similar hexagonal lattice symmetry and small and achievable lattice mismatch, and a total of six possible different stacking configurations for each vdWH were modelled. For all six different stacking configurations considered, we investigated the binding energy using *E*_b_ = *E*_MX−MX_ − (*E*_MX_ + *E*_MX_), where *E*_MX−MX_ is the total energy of the vdWHs and *E*_MX_ + *E*_MX_ is the energy of the individual parent monolayers. The calculated binding energy value for the most favourable stacking configuration is presented in Table 1, while their corresponding top and side views are given in Fig. 1. The calculated binding energy values for these stable stacking configurations are negative, which implies stronger interlayer vdW interactions and thermodynamic stability, confirming that the formation of MX–MX vdWHs is energetically favourable, exothermic in nature and likely to be realized experimentally.^46,47^

**Table 1:** Lattice constant, binding energy, interlayer distance, band gap (PBE and HSE06), work function and potential drop of MX–MX vdWHs

MX–MX vdWH	*a* (*a* = *b*) (Å)	*E*_b_ (eV)	*d* (Å)	*E*_g_ (eV) (HSE06)	*E*_g_ (eV) (PBE)	*∅* (eV)	Δ*V* (eV)
AgBr-AuBr	4.370	−0.385	3.150	1.245	0.203	2.907	4.534
AgBr-AuI	4.410	−0.274	3.30	1.337	0.446	3.068	5.125
AgBr-CuI	4.290	−0.189	3.10	2.495	1.172	2.826	4.142
AgCl-AuBr	4.270	−0.332	3.250	0.634	0.091	2.372	1.125
AgCl-AuI	4.420	−0.291	3.150	1.774	0.769	2.533	2.859
AuBr-CuI	4.20	−0.047	3.150	1.71	0.755	2.936	2.071

**Fig. 2:**
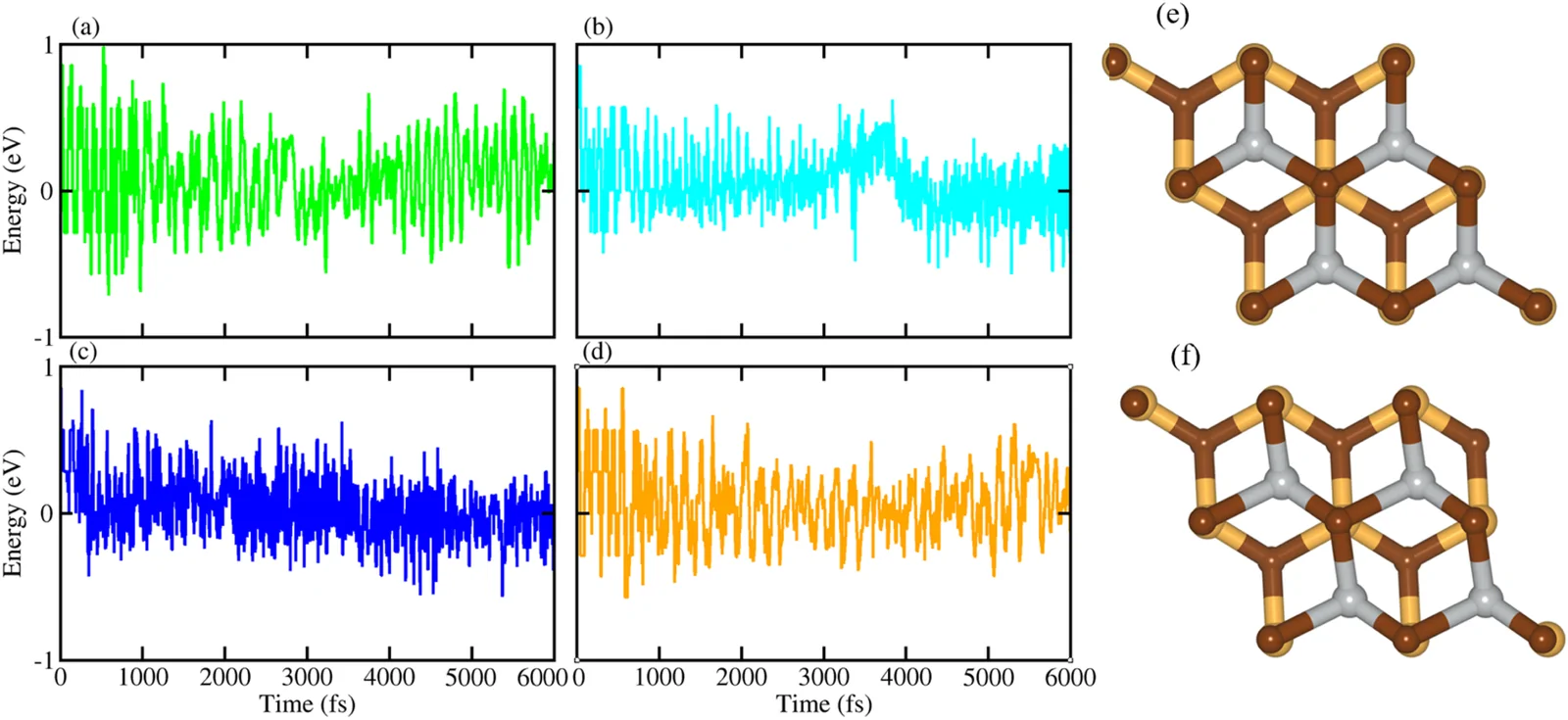
AIMD simulations of the (a) AgBr-AuBr, (b) AgBr-AuI, (c) AgBr-CuI and (d) AgCl-AuBr vdWHs. Stacking configurations of AgBr-AuBr vdWHs (e) before and (f) after heating.

To further verify the stability of these most stable stacking configurations, *ab initio* molecular dynamics (AIMD) simulations were used within the canonical (NVT) ensemble at *T* = 300 K using a total simulation time of 6 ps in the Nosé–Hoover thermostat. The total energy fluctuation as a function of time is presented in Fig. 2 for AgBr-AuBr, AgBr-AuI, AgBr-CuI and AgCl-AuBr vdWHs, which show very small amplitude fluctuations around a stable mean value, confirming a well-equilibrated system. Moreover, there is no systematic energy drift and abrupt change throughout the simulation, confirming that MX–MX vdWHs are not only theoretically stable but also practically viable for experimental realization.

**Fig. 3:**
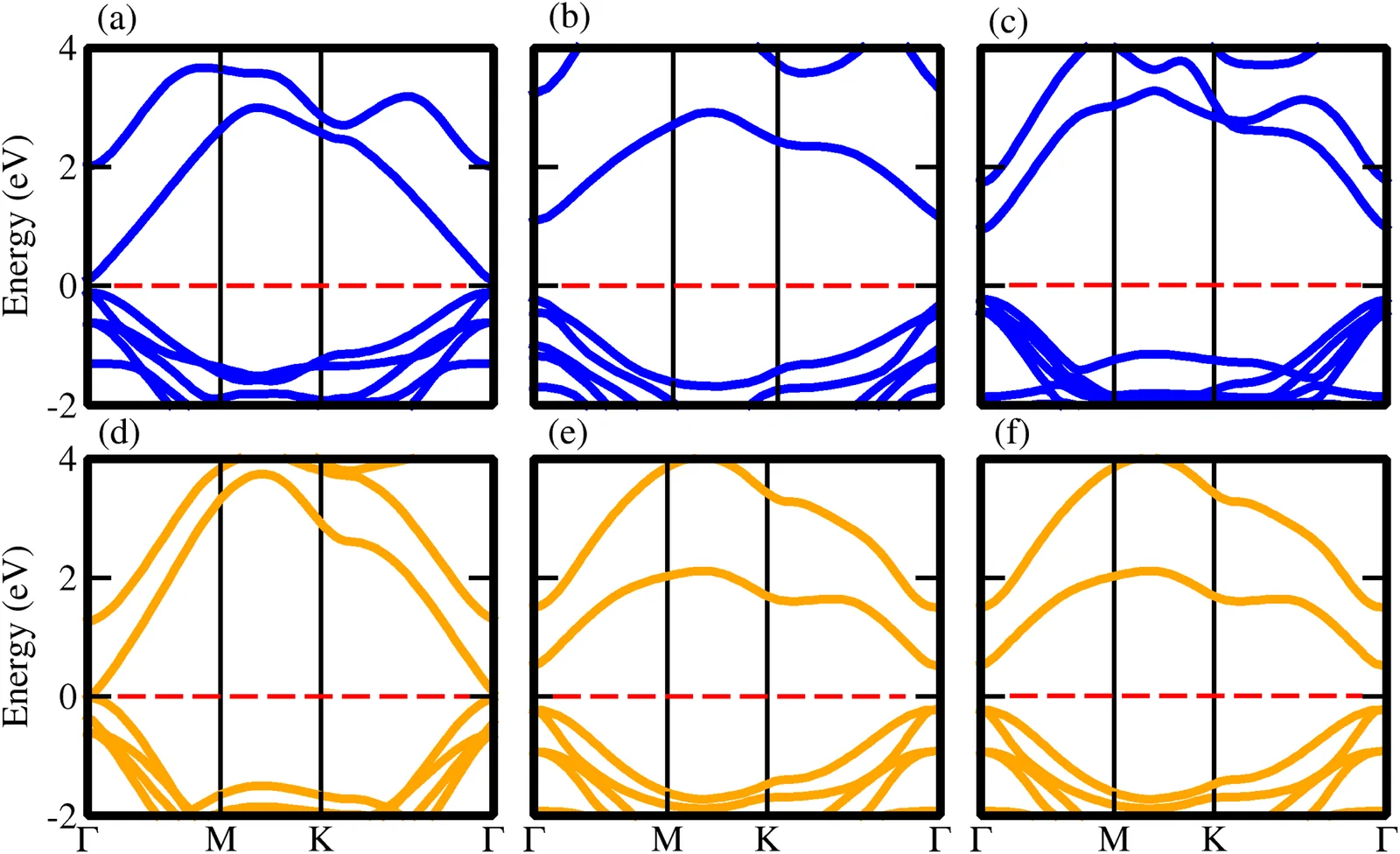
Electronic band structures calculated using the PBE functional for the (a) AgBr-AuBr, (b) AgBr-AuI, (c) AgBr-CuI, (d) AgCl-AuBr, (e) AgCl-AuI and (f) AuBr-CuI vdWHs.

**Fig. 4:**
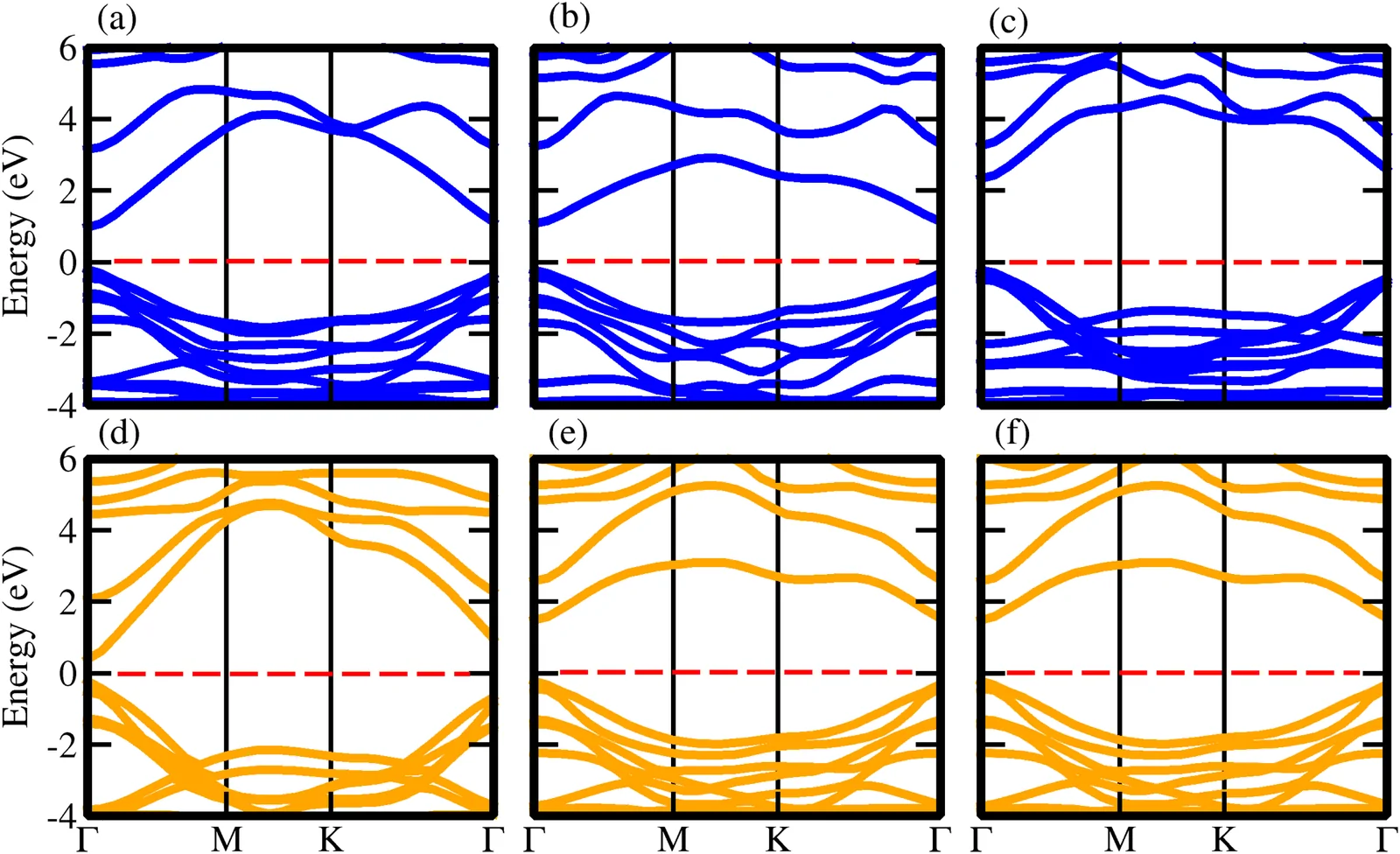
Electronic band structures calculated using the HSE06 functional for the (a) AgBr-AuBr, (b) AgBr-AuI, (c) AgBr-CuI, (d) AgCl-AuBr, (e) AgCl-AuI and (f) AuBr-CuI vdWHs.

**Fig. 5:**
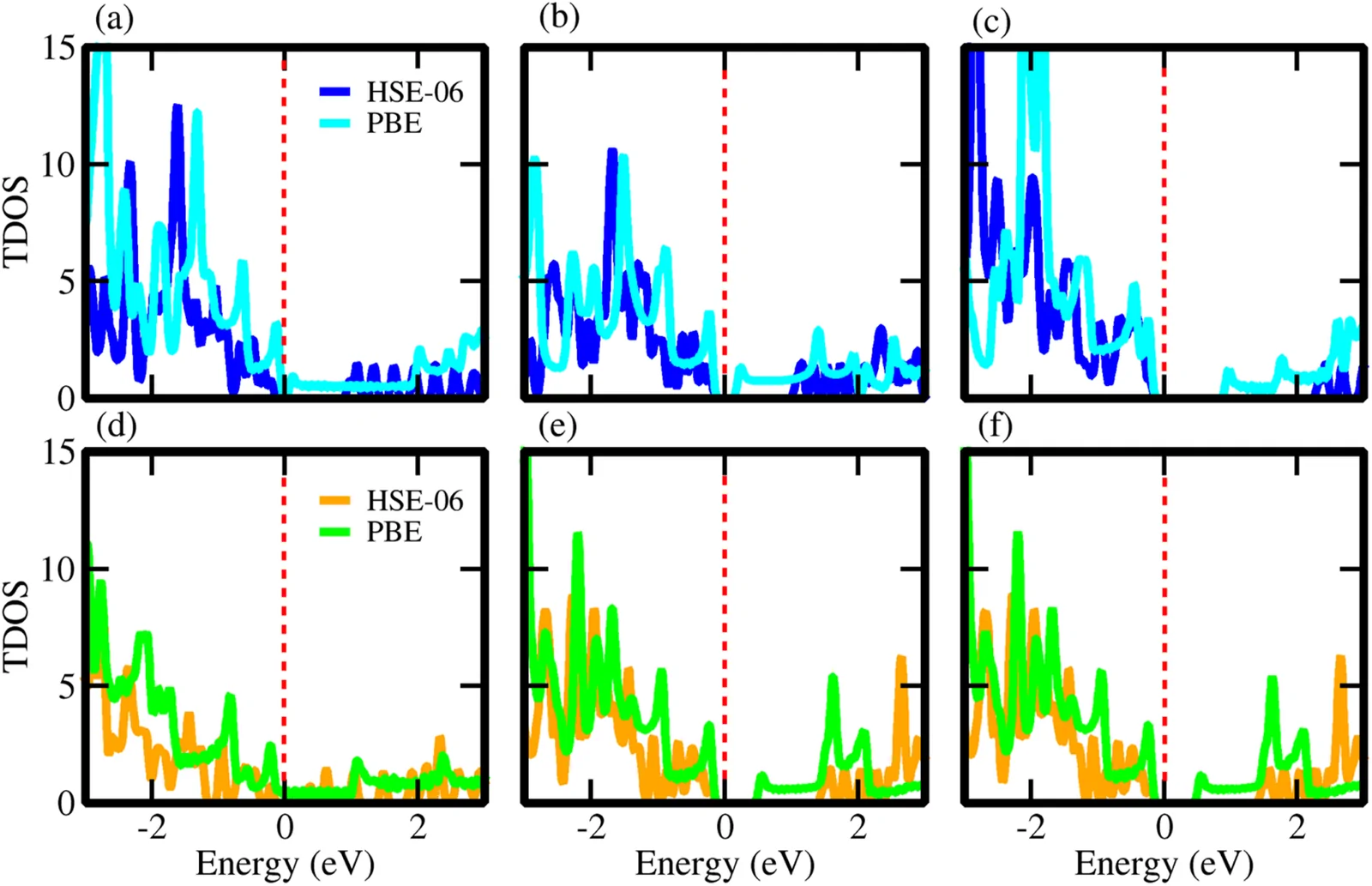
Total density of states of the (a) AgBr-AuBr, (b) AgBr-AuI, (c) AgBr-CuI, (d) AgCl-AuBr, (e) AgCl-AuI and (f) AuBr-CuI vdWHs calculated using the PBE and HSE-06 methods.

The projected electronic band structures and total densities of states (TDOS) of the MX–MX vdWHs calculated using the PBE and HSE06 functionals are given in Fig. 3 and 4, respectively, the TDOS is presented in Fig. 5, and the corresponding band gap values are listed in Table 1. The obtained electronic band structures reveal that all the investigated MX–MX vdWHs preserve a direct-gap semiconducting character, where both the CBM and VBM are localized at the same high-symmetry *Γ*-point within the first BZ. Importantly, according to the HSE06 function, MX–MX vdWHs possess band gap energies exceeding the thermodynamic threshold of 1.23 eV for photocatalytic water splitting, indicating their suitability for solar-driven hydrogen evolution applications.^48^

Additionally, the HSE06-calculated band gap values are larger than PBE-calculated band gap values, reaffirming the well-known underestimation of semiconductor band gaps within GGA schemes. We also observed that the formation of vdWHs induces substantial band-gap renormalization relative to the parent monolayers, primarily originating from interfacial electronic coupling, band-edge realignment, and weak orbital overlap near the Fermi-level region, indicating weak interlayer hybridization and vdW-dominated coupling characteristics, which are highly advantageous for controllable band engineering and tuneable nanoelectronic and optoelectronic devices.^49,50^

**Fig. 6:**
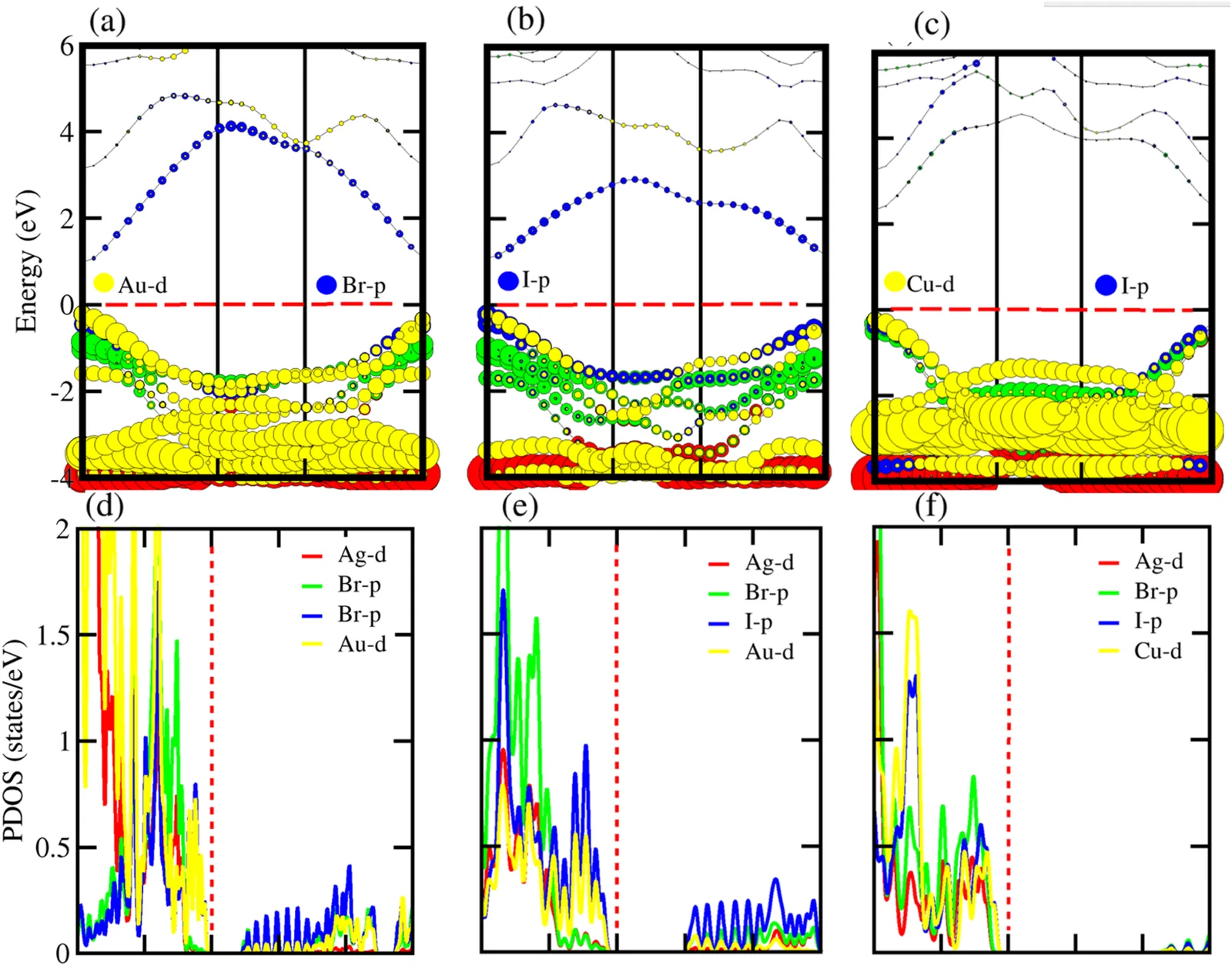
Calculated weighted band structures and PDOS of the (a and d) AgBr-AuBr, (b and e) AgBr-AuI and (c and f) AgBr-CuI vdWHs, respectively.

To further investigate the orbital and interfacial electronic states and to unequivocally ascertain the band edge alignment properties associated with the CBM and VBM, we systematically calculated the weighted band structures and partial densities of states (PDOS) of MX–MX vdWHs, as depicted in Fig. 6. Among all the investigated vdWHs, AgBr-AuI vdWHs exhibit a straddling type-I band alignment where the CBM and VBM lie within the same AuI monolayer in the I–p state, while the other vdWHs show a distinctly staggered type-II band alignment, wherein the CBM and VBM are chiefly governed by different monolayers (see Fig. 6). This type-II band alignment originates from the differences in the electron affinity and work function of the constituent monolayers, which induce interfacial charge redistribution and band offset formation. Consequently, the CBM and VBM are located on different layers, promoting the spatial separation of photogenerated electrons and holes. This suppresses electron–hole recombination, enhances charge transfer, and improves the efficiency of photocatalytic water splitting and that of optoelectronic devices, including photodetectors and solar cells. Such a pronounced spatial decoupling of frontier electronic states provides compelling evidence for intrinsically anisotropic interlayer charge redistribution across the vdW interface, establishing inherent interfacial charge separation, thereby substantially mitigating radiative electron–hole recombination processes and prolonging nonequilibrium carrier lifetimes, which is promising for high-performance photodetectors, photovoltaics, light next-generation nanoelectronics, optoelectronic platforms and photocatalytic materials.^51,52^

**Fig. 7:**
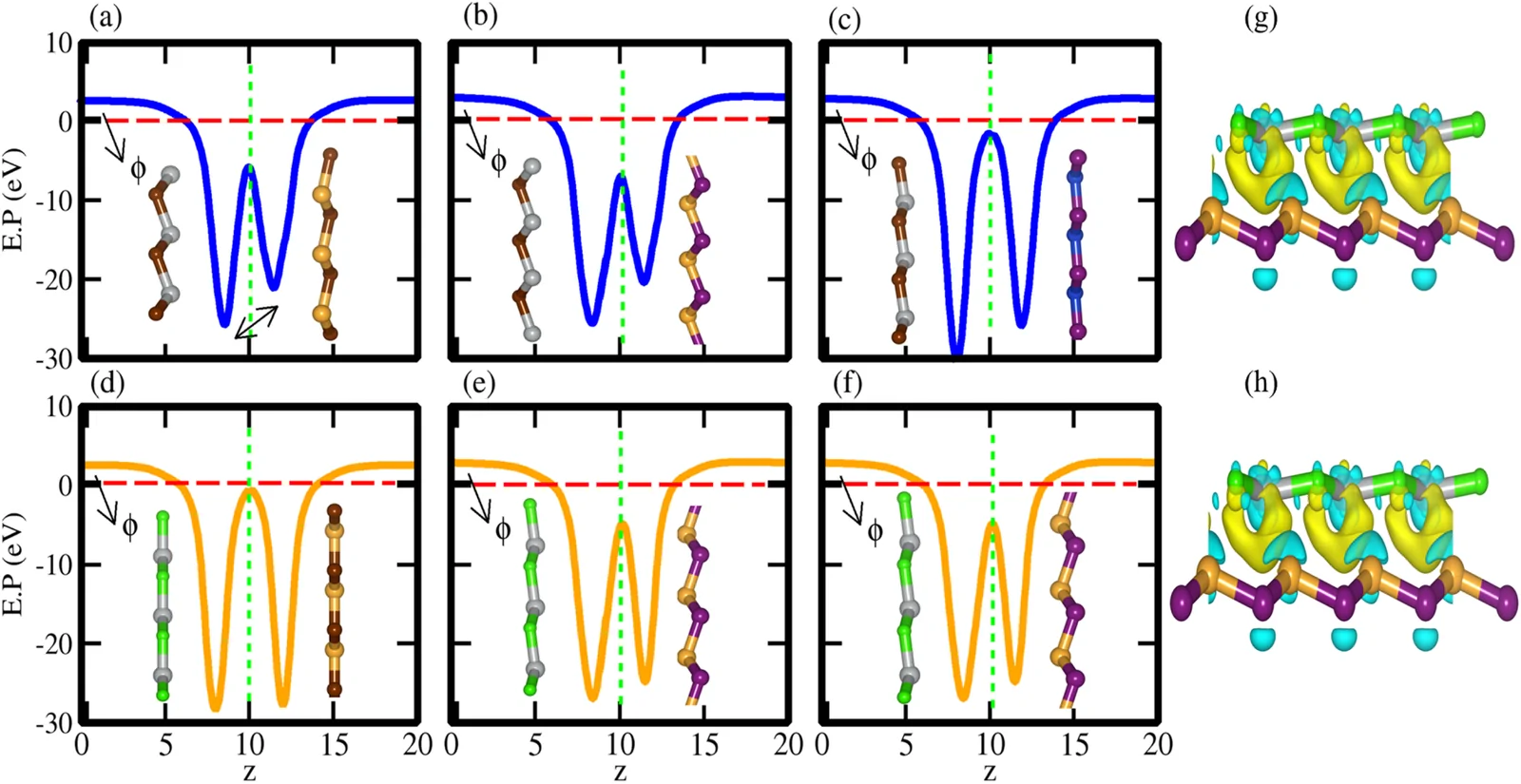
Electrostatic potentials of the (a) AgBr-AuBr, (b) AgBr-AuI, (c) AgBr-CuI, (d) AgCl-AuBr, (e) AgCl-AuI and (f) AuBr-CuI vdWHs. Charge density difference plots of (g) AgBr-AuBr and (h) AgBr-AuI vdWHs. The work function and potential drop are also mentioned in (a) to (f).

To investigate the charge transfer and p- and n-type doping in these MX–MX vdWHs, we calculated the averaged electrostatic potential, charge density difference (CDD) and Bader charge analysis using Δ*ρ* = *ρ*_MX−MX_ − *ρ*_MX_ − *ρ*_MX_, as shown in Fig. 7. The calculated Δ*ρ* reveals pronounced electron accumulation and depletion regions, where yellow and cyan isosurfaces denote charge accumulation and depletion, respectively, confirming spontaneous electron transfer from AgBr to AuBr, AgBr to AuI, AgBr to CuI, AuBr to AgCl, AuI to AgCl, and AuBr to CuI monolayers at the interface of MX–MX vdWHs, which is further confirmed by the averaged electrostatic potential difference, where AgBr, AgCl and AuBr have a higher electrostatic potential than other monolayers.

Furthermore, the substantial potential drop across these vdWHs generates remarkably strong intrinsic built-in electric fields, with potential offsets reaching as high as 6.0 eV. Such intense internal fields are highly favourable for the efficient spatial separation and directional transport of photogenerated carriers, thereby suppressing recombination kinetics and substantially enhancing optoelectronic and photovoltaic performance. Bader charge analysis shows that about 0.032 e, 0.017 e, 0.019 e, 0.026 e, 0.022 e and 0.028 e are transfer from AgBr to AuBr, AgBr to AuI, AgBr to CuI, AuBr to AgCl, AuI to AgCl, and AuBr to CuI monolayers at the interface of MX–MX vdWHs, producing p- and n-type doping, which is due to the disparity in the work functions and electronegativities of the parent monolayers, leading to significant Fermi-level realignment and electrostatic asymmetry. Here, we also investigated the work function using *Φ* = *E*_vac_ − *E*_F_, where *E*_vac_ is the vacuum energy and *E*_F_ is the Fermi energy, calculated from Fig. 7 and presented in Table 1, confirming that MX–MX vdWHs exhibit exceptional potential for advanced photocatalytic water splitting, optoelectronics and next-generation energy harvesting technologies.^53,54^

**Fig. 8:**
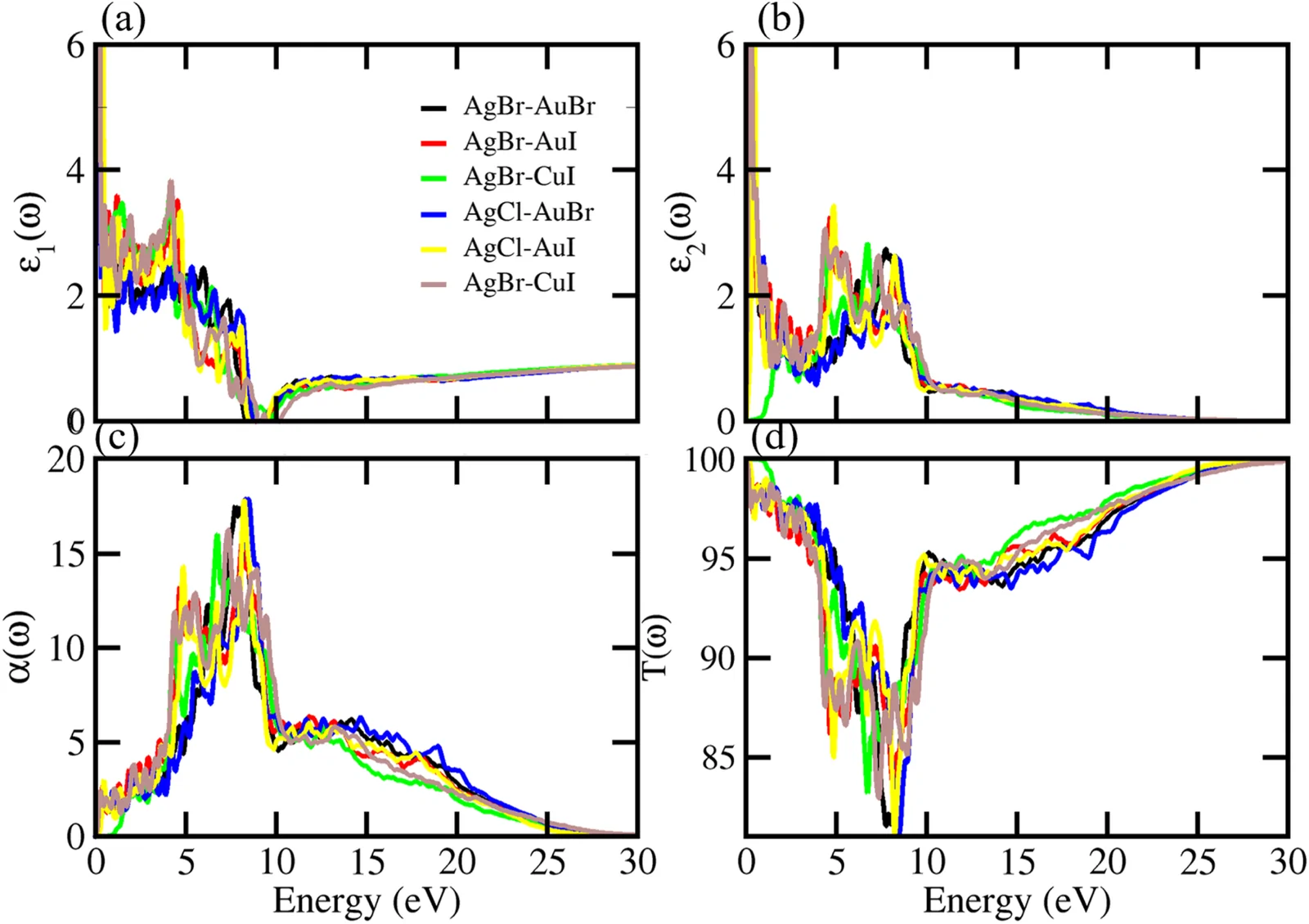
Optical response of MX–MX vdWHs: (a) real and (b) imaginary parts of the dielectric function. (c) Absorption and (d) transmission spectra. The sample legends are mentioned in the figure.

The optoelectronic properties of vdWHs are profoundly governed by their photon–matter interaction. Here, we explored the optical behaviour of MX–MX vdWHs by evaluating the frequency-dependent dielectric response, including the real and imaginary parts of the dielectric function using *ε*(*ω*) = *ε*_1_(*ω*) + *iε*_2_(*ω*), while the optical absorption was calculated by 
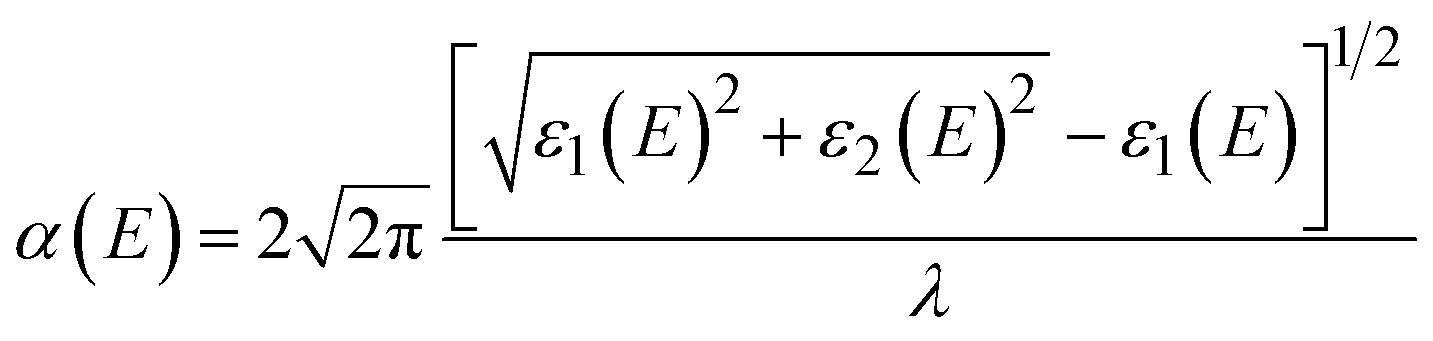
; the transmission spectra were also measured and are given in Fig. 8.^55,56^ We observed a negative value of ∼9.02–9.81 eV for the real part of the dielectric function, indicating a plasmonic resonance and metallic-like optical response. The calculated real and imaginary parts of the dielectric function of MX–MX vdWHs; the initial optical excitation peaks appear within the near-infrared (NIR) region, preceding the VR and UV regions, while the major absorption and transmission features are predominantly distributed across the VR and UR regions, indicating efficient low-energy photon interaction prior to the onset of strong visible-light absorption. The nearly linear optical response in the high-energy region is mainly attributed to the increasing contribution of higher-energy interband electronic transitions, where a higher density of electronic states results in a continuous absorption profile. This behavior is an intrinsic feature of the electronic structure of the studied heterostructures, although the selected energy range and spectral broadening used in the calculations may also contribute to the observed trend. Furthermore, all these vdWHs exhibit a remarkable absorption intensity extending from the NIR region to the UV region, confirming their ability to generate substantial photoinduced electron–hole pairs under broadband illumination, enabling efficient multispectral photoresponse spanning from the NIR and VR to UV wavelengths. Meanwhile, transmission spectra reveal a reduced transmittance within the VR region due to enhanced photon absorption, whereas a comparatively higher transmission is retained in the low-energy NIR region, making these vdWHs promising candidates for broadband photodetection, optoelectronic filtering devices, photocatalysis, transparent conductive components, and wavelength-selective photonic applications.^56–58^

**Fig. 9:**
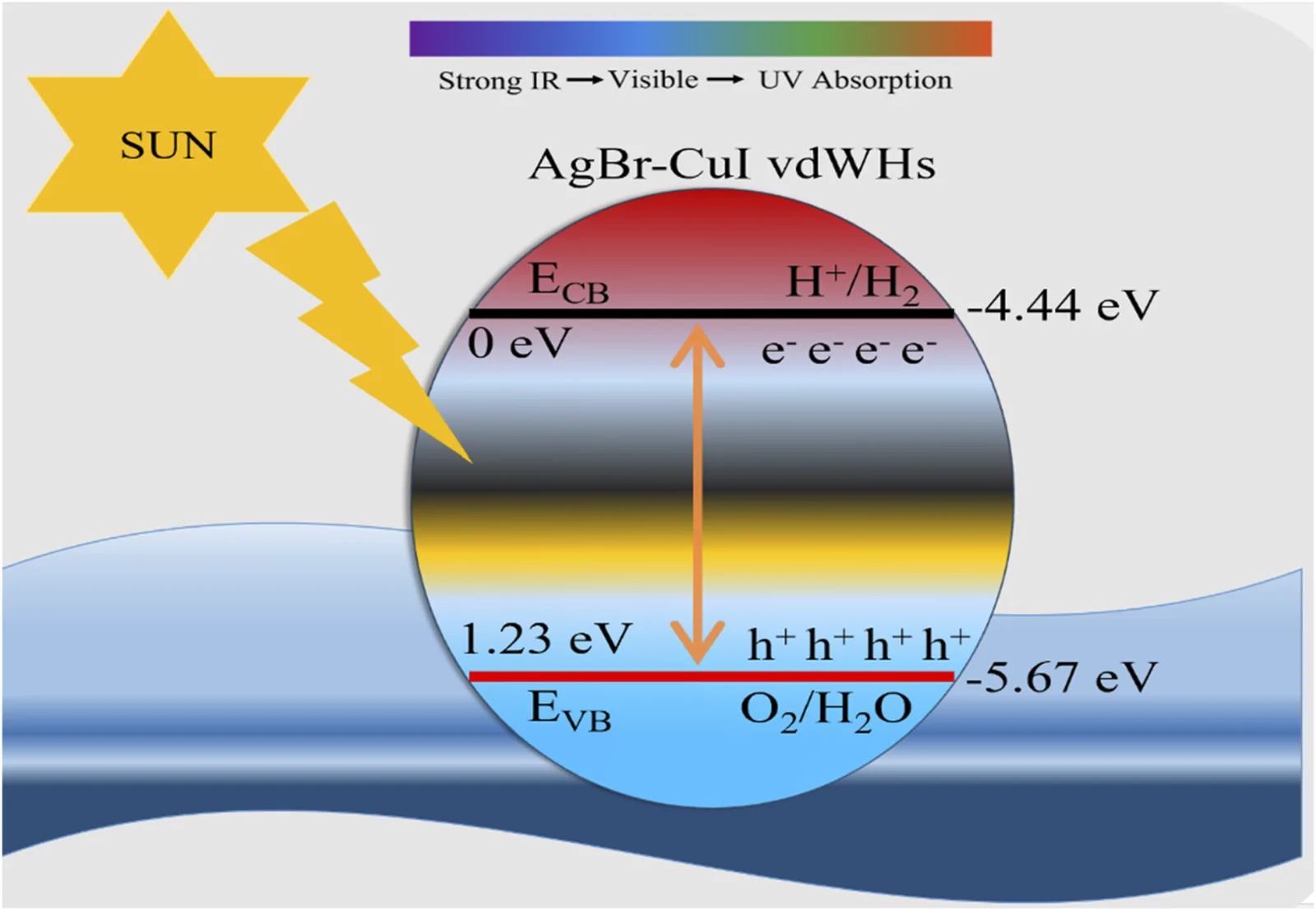
Photocatalytic response of AgBr-CuI vdWHs, with the black and red lines showing the redox potential for full water splitting.

Finally, we investigated the photocatalytic response of MX–MX vdWHs and evaluated their band edge alignment relative to the redox potentials of water. Using the HSE06 hybrid functional, *E*_VB_ and *E*_CB_ positions were referenced to the vacuum level, while the pH values were calculated using *E*_H^+^/H_2__ = 4.44 eV + pH × 0.059 eV and *E*_O_2_/H_2_O_ = 6.67 eV + pH × 0.059 eV.^59,60^ For pH = 0, the standard oxidation potential of water (H_2_O/O_2_) and reduction potential (H^+^/H_2_) are −5.67 eV and −4.44 eV, respectively, with respect to the vacuum level, and the calculated values are presented in Table 2 and illustrated for AgBr-CuI vdWHs in Fig. 9. From Table 2, one can see that at pH = 0, AgBr-CuI vdWHs possess a thermodynamically favourable electronic configuration for overall photocatalytic water splitting, wherein *E*_CB_ and *E*_VB_ appropriately straddle the hydrogen evolution (H^+^/H_2_) and oxygen evolution (O_2_/H_2_O) reaction potentials. In contrast, the remaining MX–MX vdWHs exhibit suitable *E*_VB_ positioning for the oxygen evolution reaction, indicating strong oxidation capability, while their *E*_CB_ fail to attain sufficiently negative potentials relative to the proton reduction level, thereby rendering them ineffective for hydrogen evolution. To further evaluate the photocatalytic performance under realistic operating conditions, the band edge alignment of the AgBr-CuI vdWs was analyzed over a wide pH range, as given in Table 2. According to the Nernst equation, the redox potentials shift upward by 0.059 eV per pH unit as the pH increases from 0 (acidic) to 7 (neutral) and 14 (basic/alkaline), while the band-edge positions of the vdWHs remain nearly unchanged. The calculated *E*_CB_ and *E*_VB_ of the AgBr-CuI vdWHs continue to appropriately straddle the shifted redox potentials over this pH range. These results demonstrate that the AgBr-CuI vdWHs are a promising candidate for overall photocatalytic water splitting under acidic, neutral and basic conditions, highlighting their potential for practical solar-to-hydrogen energy conversion applications. Our findings show that AgBr-CuI vdWHs are the most promising candidate for efficient overall water splitting, while the other vdWHs are primarily favourable for photocatalytic oxidation–driven processes and related photoelectrochemical applications.^2,61–64^

**Table 2:** Calculated photocatalytic response of AgBr-CuI vdWHs for pH = 0 to 14

pH value	*E*_CB_ (eV)	*E*_VB_ (eV)	pH value	*E*_CB_ (eV)	*E*_VB_ (eV)
pH = 0	−0.21807	2.27692	pH = 8	−0.69007	1.804929
pH = 1	−0.27707	2.21792	pH = 9	−0.74907	1.745929
pH = 2	−0.33607	2.15892	pH = 10	−0.80807	1.686929
pH = 3	−2.16507	0.32992	pH = 11	−0.86707	1.627929
pH = 4	−0.45407	2.04092	pH = 12	−0.92607	1.568929
pH = 05	−0.51307	1.98192	pH = 13	−0.98507	1.509929
pH = 6	−0.57207	1.92292	pH = 14	−1.04407	1.450929
pH = 7	−0.63107	1.86392	…	…	…

## Conclusion

In summary, we have systematically investigated the structural stability, interfacial electronic properties, optical response, and photocatalytic performance of MX–MX vdWHs using first-principles density functional theory calculations. The optimized vdWHs exhibit favourable energetic and dynamical stability with preserved intrinsic layered characteristics governed by weak van der Waals interactions. Electronic band structure and density of states analyses reveal that MX–MX vdWHs have type-I and type-II tuneable band gaps and pronounced interlayer electronic coupling, facilitating the efficient spatial separation of photogenerated charge carriers. Charge density difference, Bader charge and electrostatic potential analyses further confirm interfacial charge transfer and the establishment of intrinsic built-in electric fields across the heterojunctions of MX–MX vdWHs. Optical spectra demonstrate remarkable broadband optical activity extending from the near-infrared region to the ultraviolet region, accompanied by an enhanced dielectric response and strong absorption coefficients, indicating excellent light-harvesting capability. Band edge alignment calculations relative to water redox potentials show that AgBr-CuI vdWHs have a suitable energetic positioning for overall photocatalytic water splitting under acidic conditions, while the other vdWHs exhibit a favourable oxidation capability but an insufficient reduction potential for complete water splitting. These findings establish MX–MX vdWHs as a promising candidate for solar-driven hydrogen evolution, photocatalysis, and next-generation broadband optoelectronic and photoelectrochemical devices.

## Conflicts of interest

There are no conflicts to declare.

## Data Availability

The data that support the findings of this study are available from the corresponding author upon request.
